# Jamming precoding in AF relay-aided PLC systems with multiple eavessdroppers

**DOI:** 10.1038/s41598-024-58735-y

**Published:** 2024-04-09

**Authors:** Zhengmin Kong, Jiaxing Cui, Li Ding, Tao Huang, Shihao Yan

**Affiliations:** 1 School of Electrical Engineering and Automation, Wuhan University, Wuhan, 430072 China; 2 College of Science and Engineering, James Cook University, Smithfield, QLD 4878 Australia; 3 School of Science, Edith Cowan University, Joondalup, WA 6027 Australia

**Keywords:** Information technology, Electrical and electronic engineering

## Abstract

Enhancing information security has become increasingly significant in the digital age. This paper investigates the concept of physical layer security (PLS) within a relay-aided power line communication (PLC) system operating over a multiple-input multiple-output (MIMO) channel based on MK model. Specifically, we examine the transmission of confidential signals between a source and a distant destination while accounting for the presence of multiple eavesdroppers, both colluding and non-colluding. We propose a two-phase jamming scheme that leverages a full-duplex (FD) amplify-and-forward (AF) relay to address this challenge. Our primary objective is to maximize the secrecy rate, which necessitates the optimization of the jamming precoding and transmitting precoding matrices at both the source and the relay while adhering to transmit power constraints. We present a formulation of this problem and demonstrate that it can be efficiently solved using an effective block coordinate descent (BCD) algorithm. Simulation results are conducted to validate the convergence and performance of the proposed algorithm. These findings confirm the effectiveness of our approach. Furthermore, the numerical analysis reveals that our proposed algorithm surpasses traditional schemes that lack jamming to achieve higher secrecy rates. As a result, the proposed algorithm offers the benefit of guaranteeing secure communications in a realistic channel model, even in scenarios involving colluding eavesdroppers.

## Introduction

The power line channel has gained significant attention in the realm of communication networks due to its utilization of existing power line infrastructure, thus avoiding the need for additional infrastructure deployment^1^. Power line communication (PLC) has established itself as a mature technology, finding applications in various domains such as indoor, outdoor, and in-vehicle communication systems^2,3^. Meanwhile, PLC system in the home automation^4^, smart grid^5–7^, smart city^8^ and remote sensing^9^ and other fields, PLC is a strong competitor of wireless communication system. In addition, the application of PLC is not limited to the above fields, in the vehicle^10,11^, aviation equipment^12^, ship^13,14^ and train^15^ communication environment, PLC has the value of application. However, the transmission of high-frequency signals over power lines, which were not originally designed for communication purposes, poses challenges in terms of considerable attenuation over long distances. In addition to attenuation, other detrimental factors such as multipath effects resulting from impedance mismatching, distortion caused by impulsive noise^16–18^, and coupling loss (when PLC is connected to the electric power grid) further degrade the quality of data transmission in power line system^19^.

In addition to the aforementioned challenges faced by power line communication (PLC), the strict limitations on transmit power spectral density imposed by electromagnetic compatibility regulations further hinder its coverage capabilities. These limitations overlook the negative factors affecting PLC, thereby constraining its potential for reliable and high-capacity communication over long distances^20^. As a result, achieving robust and efficient PLC under unpredictable channel conditions becomes even more challenging. To overcome these limitations and enhance the reliability and reach of PLC networks, researchers have explored the application of relay-aided communication^21^. By leveraging relay nodes, which serve as intermediaries between the source and destination, relay-aided PLC offers promising opportunities for long-distance transmission in the high-frequency band^22^.

Previous research efforts have made significant strides in the development of long-distance transmission techniques for power line communication (PLC) systems employing relay nodes^23–26^. However, it is important to note that the majority of these studies primarily concentrate on the decode-and-forward (DF) relaying protocol^25,26^, with only a limited number exploring amplify-and-forward (AF) relay-aided PLC systems^24^. In comparison to DF relaying, AF approaches utilize simpler relay nodes that do not necessitate additional time for decoding, quantization, or digital signal processing. Through the straightforward process of amplifying and forwarding the received signals, AF relays can reduce the overall end-to-end transmission delay. Consequently, the performance of PLC systems based on AF relaying schemes has become an intriguing area of investigation, warranting further exploration.

The establishment of a reliable power line communication system necessitates the improvement of coverage and the guarantee of data rates, both of which are challenging due to power and bandwidth limitations^27^. In addition to the introduction of relay nodes, one practical approach to enhance data rates within a given channel quality is to improve spectral efficiency. In this regard, in-band full-duplex (IBFD) technology has showed up as a viable solution, originally explored in the realm of wireless communication^28–31^. IBFD enables simultaneous transmission and reception of signals within the same frequency band^32^. Furthermore, the application of IBFD can enhance the overall relaying capacity of a multi-hop network by enabling full-duplex relaying. This approach mitigates the repeating delays associated with each relay node, effectively doubling the data throughput^33^. IBFD has recently garnered significant attention in the context of PLC^34^. As a result, the development of IBFD technology holds great promise in enhancing the performance of long-distance PLC systems^35^.

Another significant issue in communication is to enhance security against potential negative factors^36,37^. The cheap and ubiquitous power line system is not perfect. How to enhance the security of the communication system based on power line is a serious problem with the electromagnetic radiation in the power line and the existence of malicious wired users^38,39^. Despite the incorporation of relays and in-band full duplex (IBFD) communication, long-distance power line communication (PLC) systems are inherently more vulnerable to security risks compared to conventional wireless communication. These vulnerabilities arise from factors such as impedance mismatch and non-Gaussian noise, which are characteristic of power line channels. Furthermore, in a relay-aided PLC system, the presence of malicious users who can potentially eavesdrop on messages transmitted between the source and relay nodes poses significant security threats. The security of such systems is further compromised by imperfect channel state information (CSI), which can deteriorate the overall security posture. It is important to note that power line channels and wireless channels share similarities, including frequency selectivity, frequency-dependent attenuation, and an open nature that allows any wireless or PLC device to intercept the exchanged messages. Consequently, both types of communication channels are susceptible to exploitation by malicious users^40,41^. In light of these shared vulnerabilities, it becomes possible and necessary to leverage well-investigated wireless communication technologies and security mechanisms in the context of PLC. Existing research has already demonstrated the feasibility and importance of applying established wireless communication technologies to enhance the security of PLC systems^24,42^.

The security of power line communication (PLC) can be achieved through two primary approaches: cryptographic protocols and physical layer security (PLS). While cryptographic protocols are effective in securing data transmission, PLS leverages the quality of the channel to protect against eavesdropping attacks. Compared to cryptography-based methods, PLS techniques have lower complexity and have attracted recent research interests^43^. The concept of PLS was initially introduced in the 1970s^44–46^ and has since undergone extensive research. Initially, studies focused on the degraded wiretap channel^44^, followed by investigations into the nondegraded wiretap channel^45,46^. More recently, research has delved into the analysis of fading wiretap channels^47,48^ and multiple-input–multiple-output (MIMO) wiretap channels^49–51^. PLS has also found applications in other communication scenarios, such as fiber optical networks^52^. In the context of PLC, research efforts have explored the application of PLS in different system configurations. Studies initially concentrated on PLS for single-input, single-output (SISO) PLC systems^51^, followed by investigations into PLS techniques for MIMO-based PLC systems^53^.

At present, there are some related fields of research as showed in Table 1. For example, a comprehensive study examines the ergodic secrecy achievable rate of an in-home system in the presence of an adjacent malicious wireless device^54^. Subsequently, a separate investigation analyzes the effective secrecy throughput utilizing an experimental dataset^55^. In order to enhance security, a scheme is proposed that incorporates artificial noise to improve the hybrid channel^56^. Furthermore, the research explores the PLS of cooperative relaying PLC systems under the presence of an eavesdropper^57^. To address channel noise, a scheme based on full-duplex communication with artificial noise injection is introduced^58^. Additionally, the research delves into the analysis of secrecy rates for a MIMO system, employing the IBFD jamming technique to secure the transmitted data^59^. The effective secrecy throughput is studied under the scenario of passive colluding wireless eavesdroppers, considering both in-home and broadband PLC systems^60^. Furthermore, PLS in the presence of passive eavesdropping is investigated, taking into account the impact of Bernoulli–Gaussian impulsive noise^61^. Finally, the study provides a thorough analysis of secrecy rate, secrecy outage probability, and secrecy capacity of a broadband system^62^.

**Table 1:** Overview of the existing literature.

Contributions	This work	^54^	^55^	^56^	^57^	^58^	^60^	^61^	^59^	^62^
Log-normal channel model	MK model	$\checkmark$	$\checkmark$	$\checkmark$	$\checkmark$	$\checkmark$	$\checkmark$	$\checkmark$		$\checkmark$
Bernoulli–Gaussian impulsive noise	$\checkmark$							$\checkmark$		$\checkmark$
Imperfect CSI	$\checkmark$		$\checkmark$	$\checkmark$	$\checkmark$			$\checkmark$	$\checkmark$	$\checkmark$
Multi-hop system	$\checkmark$				$\checkmark$			$\checkmark$		
Relay	AF				AF			AF		
Multiple Eves	$\checkmark$			$\checkmark$		$\checkmark$	$\checkmark$			
Proposing scheme	$\checkmark$			$\checkmark$	$\checkmark$	$\checkmark$	$\checkmark$		$\checkmark$	$\checkmark$

To the best of our knowledge, there is currently a research gap regarding relay-aided PLS in the presence of multiple eavesdroppers. Motivated by previous studies on cooperative precoding to enhance channel quality for legitimate users^63–65^ and cooperative jamming to impair channel quality for unauthorized users in wireless communication^66–68^, we propose a novel cooperative jamming and precoding PLS scheme for IBFD DF relay-aided PLC systems. Unlike other studies assuming perfect channel information^69,70^, our proposed approach takes into account imperfect channel information and aims to design the precoding matrices at the legitimate nodes. The objective is to maximize the secrecy rate of AF relay-aided PLC systems with imperfect channel information in the presence of multiple eavesdroppers. To achieve this, we utilize an efficient BCD algorithm to iteratively optimize the precoding matrices. By jointly optimizing these matrices, we aim to enhance the secrecy rate by leveraging the cooperative capabilities of the relay and introducing intentional jamming to disrupt the eavesdroppers’ reception. The proposed scheme addresses the challenges posed by multiple eavesdroppers and imperfect channel information, which are critical considerations in practical PLC systems.

To ensure the proposed scheme accurately models real-world power line channels, this paper conducts a characterization of the statistical MIMO PLC channel based on an analysis of a set of experimental field measurements^71^. The analysis takes into account various factors that impact data transfer, including fading effects, multipath propagation, and signal frequency. In addition, the noise in PLC is modeled as Bernoulli–Gaussian impulsive noise^72^. Previous research on PLS has considered different scenarios involving imperfect knowledge of CSI, ranging from passive eavesdroppers with unknown CSI^55–57,61,62^ to those with estimation errors^59,73^. We consider a system with globally imperfect channels to provide a more comprehensive and realistic approach. In this scenario, all CSIs are partially known by the legitimate nodes in the PLC system due to channel estimation errors. Furthermore, we extend the study from a single-eavesdropper scenario to a multiple-eavesdropper scenario, considering two types of eavesdropping scenarios: non-colluding and colluding. In the non-colluding scenario, the eavesdroppers operate independently and do not share information, while in the colluding scenario, all eavesdroppers collaborate to intercept the legitimate transmission. Specifically, we investigate the severe colluding case to gain deeper insights into the security performance of the proposed scheme.

The subsequent sections of this paper are organized as follows. “System model” presents a detailed description of the system model. In “Simulation and results”, the proposed optimization problem is proved to be solvable by a series of transformations. net section showcases the numerical results obtained from the proposed scheme. Finally, “Conclusion” concludes the findings and provides insights for future research directions.

Notations: To simplify the formulation, we denote ${\textbf{A}}{{\textbf{A}}^H}{\text { as }}{{\textbf{A}}^K}$ and the $\text {vec}(\textbf{A} )$ denotes the vectorization of a matrix A.

## System model

**Figure 1:**
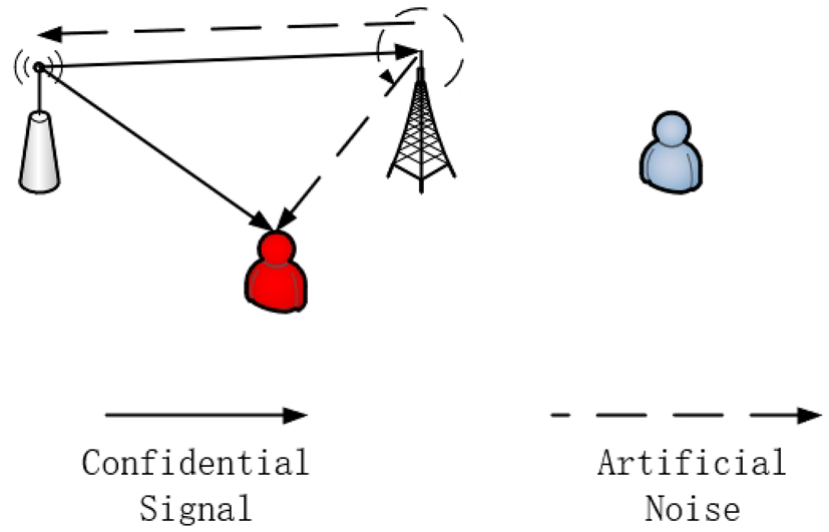
Phase 1 in PLC system.

**Figure 2:**
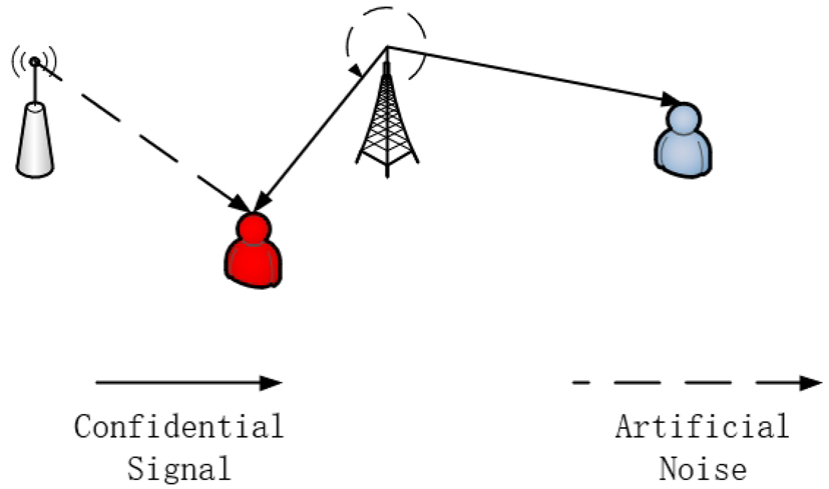
Phase 2 in the PLC system.

We consider a secure transmission system as shown in Figs. 1 and 2, where a source tries to transmit confidential information to legitimate users via an FD relay in the presence of multiple eavesdroppers eavesdropping in different time phases. More specifically, we assume all the eavesdroppers can be divided into two sets by their eavesdropping time. The first one can only eavesdrop on messages in the first time phase and the second one can only eavesdrop messages in the second time phase. Because the relay runs in the full-duplex model, self-interference should be involved. In addition, considering the huge attenuation of signals over long distances in the PLC system, the direct link from the source to the legitimate users can be ignored. In the system, the source(S), the relay(R) and the users(D) are involved ${N_S}$, ${N_R}$ and ${N_D}$ ports, respectively. Two sets of eavesdroppers are equivalent to two multiple-ports eavesdroppers (${E_1}$ and ${E_2}$). Here, both sets of eavesdroppers are equipped with ${N_E}$ ports.

In the system, channels are described by channel transfer function (CTF) ${\textbf{H}}_{ij,k}$ as the matrix of coefficients, where *i*, *j* and *k* denote the transmitter, receiver and transmission time phases, respectively. Note that ${{\textbf{H}}_{RR,1}}$ refers to the self-interference matrix , and ${\textbf{H}}_{ij,k}$ stays constant in the transmission process. In this paper, considering the multipath effect, frequency-selective effect, and time delay of power lines, this paper adopts the MK model^71^ to model the channel, with channel noise characterized as a Bernoulli–Gaussian pulse noise. Due to the imprecision of channel estimation/feedback and the stealthiness of eavesdroppers, it is challenging for the transmitter to obtain accurate channel state information between the receiver and the transmitter. Therefore, we consider all channels to be imperfect channels.

The imperfect channels are described by the deterministic uncertainty model:

1 $$\begin{aligned} {{\textbf{H}}_{ij,k}} \!\!\in \!\!{{{\mathcal {H}}}_{ij,k}}\!\!= \left\{ {{{\textbf{H}}_{ij,k}}|{{\textbf{H}}_{ij,k}} \!\!= {{\overline{\textbf{H}}}_{ij,k}}\!\! + {\Delta _{ij,k}},\left\| {{\Delta _{ij,k}}} \right\| \le {\delta _{ij,k}}} \right\} , \end{aligned}$$

where ${{\Delta }_{ij,k}}$ and ${{\overline{\textbf{H}}}_{ij,k}}$ denote the CTF error and the mean CTF, respectively.

The PLC system is operated over two-time phases with the relay in the AF model. In the two time slots, confidential information is transmitted via a source and a relay. While one of the legitimate nodes propagates the information forward, the other sends jamming signal to deal with possible eavesdroppers. By considering the possible worst-case scenario, the model introduces two groups of eavesdroppers who eavesdrop on two time slots respectively and considers the effect of self-interference at the relay.

In the first time phase, the source broadcasts confidential messages to the relay and the messages are inevitably eavesdropped by ${E_1}$. More accurately, the confidential messages are modeled as symbols ${\textbf{S}} \in {{\mathcal {C}}}{{\mathcal {N}}}({\textbf{0}},1)$ mapped by vector ${\textbf{W}} \in {{\mathbb {C}}^{{N_S} \times 1}}$:

2 $$\begin{aligned} {{\textbf{X}}_{_S}} = {\textbf{WS}}, \end{aligned}$$

Next, we consider the messages emitted by the relay which only emits jamming:

3 $$\begin{aligned} {{\textbf{X}}_R} = {\textbf{VZ}}, \end{aligned}$$

where ${\textbf{V}} \in {{\mathbb {C}}^{{N_R} \times 1}}$ and ${\textbf{Z}} \in \mathcal {C}\mathcal {N}({\textbf{0}},1)$ denote jamming vector and symbol.

With self-interference, the messages received by the relay can be formulated:

4 $$\begin{aligned} {{\textbf{Y}}_{R1}} = {{\textbf{H}}_{SR,1}}{\textbf{WS}} + {{\textbf{H}}_{RR,1}}{\textbf{VZ}} + {{\textbf{n}}_{R1}}, \end{aligned}$$

where ${{\textbf{n}}_{R1}}$ is actually PLC noise based on the Bernoulli–Gaussian noise model at the relay.

Meanwhile, ${E_1}$ receives the messages from both the source and the relay:

5 $$\begin{aligned} {{\textbf{Y}}_{E1}} = {{\textbf{H}}_{SE,1}}{\textbf{WS}} + {{\textbf{H}}_{RE,1}}{\textbf{VZ}} + {{\textbf{n}}_{E1}}, \end{aligned}$$

where ${{\textbf{n}}_{E1}}$ is Bernoulli–Gaussian noise at ${E_1}$.

In the second time phase, the source emits the jamming precoded from the relay:

6 $$\begin{aligned} {{\textbf{X}}_{S2}} = {\textbf{AZ}}, \end{aligned}$$

Meanwhile, the relay is working in the AF mode so it amplifies and forwards the messages it received to both the users and ${E_2}$:

7 $$\begin{aligned} {{\textbf{X}}_{R2}}{\text { = }}{\textbf{G}}{{\textbf{Y}}_{R1}} = {\textbf{G}}({{\textbf{H}}_{SR,1}}{\textbf{WS}} +{{\textbf{H}}_{RR,1}}{\textbf{VZ}} + {{\textbf{n}}_{R1}}), \end{aligned}$$

where ${\textbf{G}} \in {{\mathbb {C}}^{{N_R} \times {N_R}}}$ denotes the amplifying matrix.

Because of the distance, the jamming from the source will not interrupt users but ${E_2}$, i.e.

8 $$\begin{aligned} {{\textbf{Y}}_{E2}} = {{\textbf{H}}_{SE,2}}{\textbf{AZ}}+ {{\textbf{H}}_{RE,2}}{\textbf{G}}({{\textbf{H}}_{SR,1}}{\textbf{WS}}+ {{\textbf{H}}_{RR,1}}{\textbf{VZ}} + {{\textbf{n}}_{R1}}) + {{\textbf{n}}_{E2}} \end{aligned}$$

9 $$\begin{aligned} {{\textbf{Y}}_D} = {{\textbf{H}}_{RD,2}}{{\textbf{X}}_{R2}} + {{\textbf{n}}_D}= {{\textbf{H}}_{RD,2}}{\textbf{G}}({{\textbf{H}}_{SR,1}}{\textbf{WS}} + {{\textbf{H}}_{RR,1}}{\textbf{VZ}} + {{\textbf{n}}_{R1}}) + {{\textbf{n}}_D}, \end{aligned}$$

where ${{\textbf{n}}_{D}}$ is Bernouil–Gaussia noise at the users and ${{\textbf{n}}_{E2}}$ is Bernouil–Gaussia noise at ${E_2}$.

Above all, we can calculate the signal-to-noise ratio (SNR) at all the receivers.

10 $$\begin{aligned} {{{{{ \gamma }}_D} = {({{\textbf{H}}_{RD,2}}{\textbf{G}}{{\textbf{H}}_{SR,1}}{\textbf{W}})^K}{\textbf{Q}}_D^{ - 1}, \hspace{5.0pt}{\textrm{where }} \hspace{5.0pt}{{\textbf{Q}}_D} = {({{\textbf{H}}_{RD,2}}{\textbf{G}}{{\textbf{H}}_{RR,1}}{\textbf{V}})^K} + \sigma _R^2{({{\textbf{H}}_{RD,2}}{\textbf{G}})^K} + \sigma _D^2{\textbf{I}}}} \end{aligned}$$

11 $$\begin{aligned} {{{{\mathbf{\gamma }}_{E1}} = {({{\textbf{H}}_{SE,1}}{\textbf{W}})^K}{\textbf{Q}}_{E1}^{ - 1}, \hspace{5.0pt}{\textrm{where}} \hspace{5.0pt}{{\textbf{Q}}_{E1}} = {({{\textbf{H}}_{RE,1}}{\textbf{V}})^K} + \sigma _E^2{\textbf{I}}}} \end{aligned}$$

12 $$\begin{aligned} {{{{\mathbf{\gamma }}_{E2}} = {({{\textbf{H}}_{RE,2}}{\textbf{G}}{{\textbf{H}}_{SR,1}}{\textbf{W}})^K}{\textbf{Q}}_{E2}^{ - 1},\hspace{5.0pt}{\textrm{where }} \hspace{5.0pt}{{\textbf{Q}}_{E2}} = {({{\textbf{H}}_{SE,2}}{\textbf{A}})^K} + {({{\textbf{H}}_{RE,2}}{\textbf{G}}{{\textbf{H}}_{RR,1}}{\textbf{V}})^K} + \sigma _R^2{({{\textbf{H}}_{RE,2}}{\textbf{G}})^K} + \sigma _E^2{\textbf{I}}}} \end{aligned}$$

and $\sigma _i^{}$ is the amplitude of the corresponding Bernouil–Gaussia noise ${{\textbf{n}}_i}$.

The achievable rate of the legitimate users is as follows:

13 $$\begin{aligned} {{{R_D} = \log \left| {{\textbf{I}} + {{\mathbf{\gamma }}_D}} \right| }}, \end{aligned}$$

However, the situation for eavesdropping is more complicated, because the eavesdroppers can collude or not. In the colluding case, the eavesdroppers can utilize maximum ratio combining (MRC) to combine their received information. In this typical collusion strategy, the eavesdropping SNR is the sum of all the eavesdroppers. So the achievable rate of the eavesdroppers is as follows:

14 $$\begin{aligned} {{{R_{E}} = {\log \left| {{\textbf{I}} + {{\mathbf{\gamma }}_{E1}} + {{\mathbf{\gamma }}_{E2}}} \right| } }} \end{aligned}$$

### Jamming precoding scheme

In this work, the goal is to maximize the secrecy rate of the system. With the transmit power constraint, the optimization problem can be formulated as follows.

15 $$\begin{aligned} {{\mathop {\max }\limits _{{\textbf{W}},{\textbf{V}},{\textbf{A}},{\textbf{G}}} {\hspace{0.55542pt}} \mathop {\min }\limits _{{{\textbf{H}}_{ij,k}} \in {\mathcal {H}_{ij,k}}} {\hspace{0.55542pt}} {R_D} - {R_E}}} \end{aligned}$$

16 $$\begin{aligned}{}&{{{\text {s}}.{\text {t}}{\text {. }}{\left\| {\textbf{W}} \right\| ^2} \leqslant {P_S},\;{\left\| {\textbf{A}} \right\| ^2} \leqslant {P_S},{\left\| {\textbf{V}} \right\| ^2} \leqslant {P_R},}} \hspace{5.0pt}\text {tr}({({\textbf{G}}{{\textbf{H}}_{SR,1}}{\textbf{W}})^K} + {({\textbf{G}}{{\textbf{H}}_{RR,1}}{\textbf{V}})^K} + \sigma _R^2{{\textbf{G}}^K}) \leqslant {P_R} \quad \forall {{\textbf{H}}_{ij,k}} \in {\mathcal {H}_{ij,k}} \end{aligned}$$

Because of the high non-convexity of the function $\log \left| \cdot \right|$, the problem is hard to solve. To make the problem solvable, (15) is transformed into an equivalent form through WMMSE algorithm, which can be solved with the BCD algorithm. We first introduce WMMSE algorithm.

Lemma1^74^: Define the MSE matrix

$$\begin{aligned} \widehat{\textbf{M}}\triangleq {{(\textbf{DH}\text {-}\textbf{I})}^{K}}+\textbf{DR}{{\textbf{D}}^{H}} \end{aligned}$$

17 $$\begin{aligned} {{\begin{aligned} {}- {R_{E}}&= \log \left| {\textbf{Q}} \right| - \log \left| {{\textbf{Q}} + {{\textbf{P}}^K}} \right| \\&= \log \left| {{\textbf{I}} + \left[ {\begin{array}{*{20}{c}} {\sigma _E^{ - 2}{{({{\textbf{H}}_{RE,1}}{\textbf{V}})}^K}}&{}{\textbf{0}} \\ {\textbf{0}}&{}{\sigma _E^{ - 2}[{{({{\textbf{H}}_{SE,2}}{\textbf{A}})}^K} + {{({{\textbf{H}}_{RE,2}}{\textbf{G}}{{\textbf{H}}_{RR,1}}{\textbf{V}})}^K} + \sigma _R^2{{({{\textbf{H}}_{RE,2}}{\textbf{G}})}^K}]} \end{array}} \right] } \right| \\&\quad -\log \left| {{\textbf{I}} + \left[ {\begin{array}{*{20}{c}} {\sigma _E^{ - 2}{{({{\textbf{H}}_{RE,1}}{\textbf{V}})}^K}}&{}{\textbf{0}} \\ {\textbf{0}}&{}{\sigma _E^{ - 2}[{{({{\textbf{H}}_{SE,2}}{\textbf{A}})}^K} + {{({{\textbf{H}}_{RE,2}}{\textbf{G}}{{\textbf{H}}_{RR,1}}{\textbf{V}})}^K} + \sigma _R^2{{({{\textbf{H}}_{RE,2}}{\textbf{G}})}^K}]} \end{array}} \right] + {{\left[ {\begin{array}{*{20}{c}} {\sigma _E^{ - 1}{{\textbf{P}}_1}} \\ {\sigma _E^{ - 1}{{\textbf{P}}_2}} \end{array}} \right] }^K}} \right| \\&= \underbrace{\log \left| {{\textbf{I}} + \sigma _E^{ - 2}{{({{\textbf{H}}_{RE,1}}{\textbf{V}})}^K}} \right| }_{{C_{E1}}} + \underbrace{\log \left| {{\textbf{I}} + \sigma _E^{ - 2}[{{({{\textbf{H}}_{SE,2}}{\textbf{A}})}^K} + {{({{\textbf{H}}_{RE,2}}{\textbf{G}}{{\textbf{H}}_{RR,1}}{\textbf{V}})}^K} + \sigma _R^2{{({{\textbf{H}}_{RE,2}}{\textbf{G}})}^K}]} \right| }_{{C_{E2}}} \\&\quad +\underbrace{ - \log \left| {\underbrace{{\textbf{I}} + \left[ {\begin{array}{*{20}{c}} {\sigma _E^{ - 2}{{({{\textbf{H}}_{RE,1}}{\textbf{V}})}^K}}&{}{\textbf{0}} \\ {\textbf{0}}&{}{\sigma _E^{ - 2}[{{({{\textbf{H}}_{SE,2}}{\textbf{A}})}^K} + {{({{\textbf{H}}_{RE,2}}{\textbf{G}}{{\textbf{H}}_{RR,1}}{\textbf{V}})}^K} + \sigma _R^2{{({{\textbf{H}}_{RE,2}}{\textbf{G}})}^K}]} \end{array}} \right] + {{\left[ {\begin{array}{*{20}{c}} {\sigma _E^{ - 1}{{\textbf{P}}_1}} \\ {\sigma _E^{ - 1}{{\textbf{P}}_2}} \end{array}} \right] }^K}}_{{{\textbf{M}}_{E3}}}} \right| }_{{C_{E3}}} \\ \end{aligned}}} \end{aligned}$$

where $\textbf{R}\succ \textbf{0}$. Then we have

18 $$\begin{aligned} {{{{ - log}}\left| {\textbf{M}} \right| = \mathop {\max }\limits _{{\textbf{S}} \succ 0} {\hspace{0.55542pt}} \log \left| {\textbf{S}} \right| - {\textrm{tr}}({\textbf{SM}}) + {\textrm{tr}}({\textbf{I}}) \quad \quad \quad \log \left| {{\textbf{I}} + {{\textbf{R}}^{ - 1}}{{\textbf{H}}^K}} \right| = \mathop {\max }\limits _{{\textbf{S}} \succ 0,{\textbf{D}}} {\hspace{0.55542pt}} \log \left| {\textbf{S}} \right| - {\textrm{tr}}({\textbf{S}}\widehat{\textbf{M}}) + {\textrm{tr}}({\textbf{I}})}} \end{aligned}$$

Furthermore, auxiliary matrices ${{\textbf{S}}_{i}},{{\textbf{M}}_{i}},{{\textbf{D}}_{i}}$ are introduced to reformulate the part of $\log \left| \cdot \right|$ in the objective function in (15) as follows.

19 $$\begin{aligned} {{R}_{D}}=\underset{{{\textbf{S}}_{D}}\succ 0,{{\textbf{D}}_{D}}}{\mathop {\max }}\,\log \left| {{\textbf{S}}_{D}} \right| -\text {tr}({{\textbf{S}}_{D}}{{\textbf{M}}_{D}})+\text {tr}(\textbf{I}) \end{aligned}$$

where

20 $$\begin{aligned} {{{{\textbf{M}}_D} = {({{\textbf{D}}_D}{{\textbf{H}}_{RD,2}}{\textbf{G}}{{\textbf{H}}_{SR,1}}{\textbf{W}} - {\textbf{I}})^K} + {{\textbf{D}}_D}{{\textbf{Q}}_D}{\textbf{D}}_D^H}} \end{aligned}$$

However, (14) is hard to explicit the Lemma1 directly. As a result, we need to transform (14) in a more compatible form.

21 $$\begin{aligned} {{\log \left| {{\textbf{I}} + {{\mathbf {\gamma }}_{E1}} + {{\mathbf {\gamma }}_{E2}}} \right| = \log \left| {{\textbf{I}} + {{\textbf{P}}^K}{{\textbf{Q}}^{ - 1}}} \right| }} \end{aligned}$$

where

$$\begin{aligned}{\textbf{Q}} = \left[ {\begin{array}{*{20}{c}} {{{\textbf{Q}}_{E1}}}&{}{\textbf{0}}\\ {\textbf{0}}&{}{{{\textbf{Q}}_{E2}}} \end{array}} \right] ,{\textbf{P}} = \left[ {\begin{array}{*{20}{c}} {{{\textbf{P}}_1}}\\ {{{\textbf{P}}_2}} \end{array}} \right] = \left[ {\begin{array}{*{20}{c}} {{{\textbf{H}}_{SE,1}}{\textbf{W}}}\\ {{{\textbf{H}}_{RE,2}}{\textbf{G}}{{\textbf{H}}_{SR,1}}{\textbf{W}}} \end{array}} \right] \end{aligned}$$

Then we have (17), where ${R_{E}}$ are divided as ${C_{E1}}$, ${C_{E2}}$ and ${C_{E3}}$ for subsequent transformation. Thus, in order to formulate the achievable rate of the eavesdroppers in the colluding case, ${C_{E1}}$ and ${C_{E2}}$ is equivalent to

22 $$\begin{aligned} {{{C_{E1}} = \mathop {\max }\limits _{{{\textbf{S}}_{E1}} \succ 0,{{\textbf{D}}_{E1}}} \log \left| {{{\textbf{S}}_{E1}}} \right| - {\text {tr}}({{\textbf{S}}_{E1}}{{\textbf{M}}_{E1}}) + {\text {tr}}({\textbf{I}})}} \end{aligned}$$

23 $$\begin{aligned} {{{C_{E2}} = \mathop {\max }\limits _{{{\textbf{S}}_{E2}} \succ 0,{{\textbf{D}}_{E2}}} \log \left| {{{\textbf{S}}_{E2}}} \right| - {\text {tr}}({{\textbf{S}}_{E2}}{{\textbf{M}}_{E2}}) + {\text {tr}}({\textbf{I}})}} \end{aligned}$$

where

$$\begin{aligned}    &{{\textbf{M}}_{E1}} = {({{\textbf{D}}_{E1}}{{\textbf{H}}_{RE,1}}{\textbf{V}} - {\textbf{I}})^K} + \sigma _E^2{\textbf{D}}_{E1}^K \\ & {{\textbf{M}}_{E2}} = ({{\textbf{D}}_{E21}}{{\textbf{H}}_{SE,2}}{\textbf{AX}} + {{\textbf{D}}_{E22}}{{\textbf{H}}_{RE,2}}{\textbf{G}}{{\textbf{H}}_{RR,1}}{\textbf{VX}} + \sigma _R^{}{{\textbf{D}}_{E23}}{{\textbf{H}}_{RE,2}}{\textbf{G}} - {\textbf{I}}{)^K} + \sigma _E^2\left( {{\textbf{D}}_{E21}^K + {\textbf{D}}_{E22}^K + {\textbf{D}}_{E23}^K} \right) \end{aligned}  $$

24 $$\begin{aligned} {f} \triangleq \log \left| {{{\textbf{S}}_D}} \right| - {\text {tr}}({{\textbf{S}}_D}{{\textbf{M}}_D}) + \log \left| {{{\textbf{S}}_{E1}}} \right| - {\text {tr}}({{\textbf{S}}_{E1}}{{\textbf{M}}_{E1}}) + \log \left| {{{\textbf{S}}_{E2}}} \right| - {\text {tr}}({{\textbf{S}}_{E2}}{{\textbf{M}}_{E2}}) + \log \left| {{{\textbf{S}}_{E3}}} \right| - {\textbf{tr}}({{\textbf{S}}_{E3}}{{\textbf{M}}_{E3}}) \end{aligned}$$

25 $$\begin{aligned} {{{g} \buildrel \Delta \over = \log \left| {{{\textbf{S}}_D}} \right| - {\beta _D} + \log \left| {{{\textbf{S}}_{E1}}} \right| - {\beta _{E1}} + \log \left| {{{\textbf{S}}_{E2}}} \right| - {\beta _{E2}} + \log \left| {{{\textbf{S}}_{E3}}} \right| - {\beta _{E3}}}} \end{aligned}$$

Note the decomposition $${{{\textbf{D}}_{E2}} = \left[ {\begin{array}{*{20}{c}} {{{\textbf{D}}_{E21}}}&{{{\textbf{D}}_{E22}}}&{{{\textbf{D}}_{E23}}} \end{array}} \right] }$$ and $${{\textbf{X}} = \left[ {\begin{array}{*{20}{c}} 1&{\textbf{0}} \end{array}} \right] \in {{\mathbb {C}}^{1 \times N_{R}}}}$$.

Then to solve ${C_{E3}}$, we also apply Lemma1 :

26 $$\begin{aligned} {{{C_{E3}} = \mathop {\max }\limits _{{{\textbf{S}}_{E3}} \succ 0} \log \left| {{{\textbf{S}}_{E3}}} \right| - {\textbf{tr}}({{\textbf{S}}_{E3}}{{\textbf{M}}_{E3}}) + {\textbf{tr}}({\textbf{I}})}} \end{aligned}$$

After substituting (22)–(26) into (15), the secrecy rate of the system with the colluding eavesdroppers can be rewritten as

27 $$\begin{aligned} \underset{\textbf{W},\textbf{V},\textbf{A},{\textbf{G}},{{\textbf{S}}_{i}}\succ 0,{{\textbf{D}}_{i}}}{\mathop {\max }}\,\underset{{{\textbf{H}}_{ij,k}}\in {{\mathcal {H}}_{ij,k}}}{\mathop {\min }}\,f(\textbf{W},\textbf{V},\textbf{A},{\textbf{G}},{{\textbf{S}}_{\textbf{i}}},{{\textbf{D}}_{\textbf{i}}}),\quad \text {s}.\text {t}. (16) \end{aligned}$$

where $f(\textbf{W},\textbf{V},\textbf{A},{\textbf{G}},{{\textbf{S}}_{\textbf{i}}},{{\textbf{D}}_{\textbf{i}}})$ is defined in (24).

The max-min problem and constrain ${\textrm{tr}}({({\textbf{G}}{{\textbf{H}}_{SR,1}}{\textbf{W}})^K} + {({\textbf{G}}{{\textbf{H}}_{RR,1}}{\textbf{V}})^K} + \sigma _R^2{{\textbf{G}}^K}) \le {P_R}$ can be transformed into an optimization problem by introducing constraints with slack variables ${\beta }_{i}$ as follows.

28 $$\begin{aligned} \text {tr}({{\textbf{S}}_{i}}{{\textbf{M}}_{i}})\le {{\beta }_{i}}, \forall {{\textbf{H}}_{ij,k}}\in {{\mathcal {H}}_{ij,k}} \end{aligned}$$

The problem (27) can be further transformed as

29 $$\begin{aligned} {{\mathop {\max }\limits _{{\textbf{W}},{\textbf{V}},{\textbf{A}},{\textbf{G}},{{\textbf{S}}_i} \succ 0,{{\textbf{D}}_i}} {\hspace{0.55542pt}} {g}({\textbf{W}},{\textbf{V}},{\textbf{A}},{\textbf{G}},{{\textbf{S}}_{\textbf{i}}},{{\textbf{D}}_{\textbf{i}}})}}, \quad \text {s}.\text {t}. (16),\;(26) \end{aligned}$$

where $g(\textbf{W},\textbf{V},\textbf{A},{\textbf{G}},{{\textbf{S}}_{\textbf{i}}},{{\textbf{D}}_{\textbf{i}}})$ is defined in (25).

However, (25) is still convex because of the semi-infinite constraints (28). For $i=D$, $\text {tr}({{\textbf{S}}_{D}}{{\textbf{M}}_{D}})$ can be rewritten as

30 $$\begin{aligned} {\text {tr}}({{\textbf{S}}_D}{{\textbf{M}}_D}) = {\left\| {\underbrace{\left[ {\begin{array}{*{20}{c}} {{\text {vec}}({{\textbf{F}}_D}({{\textbf{D}}_D}{{\textbf{H}}_{RD,2}}{\textbf{G}}{{\textbf{H}}_{SR,1}}{\textbf{W}} - {\textbf{I}}))} \\ {{\text {vec}}({{\textbf{F}}_D}{{\textbf{D}}_D}{{\textbf{H}}_{RD,2}}{\textbf{G}}{{\textbf{H}}_{RR,1}}{\textbf{V}})} \\ {{\text {vec}}({\sigma _R}{{\textbf{F}}_D}{{\textbf{D}}_D}{{\textbf{H}}_{RD,2}}{\textbf{G}})} \\ {{\text {vec}}({\sigma _D}{{\textbf{F}}_D}{{\textbf{D}}_D})} \end{array}} \right] }_{{\phi _D}}} \right\| ^2} \end{aligned}$$

where ${\textbf{S}}_D^{} = {\textbf{F}}_D^H{\textbf{F}}_D^{}$ and the equality $\text {tr}({{\textbf{A}}^{K}})={{\left\| \text {vec}(\textbf{A}) \right\| }^{2}}$ is applied.

Especially note that for $i=E3$, to obtain similiar form as (30), ${{\textbf{F}}_{E3}}$ should be divided as

31 $$\begin{aligned} {{{\textbf{F}}_{E3}} = \left[ {\begin{array}{*{20}{c}} {{{\textbf{F}}_{E31}}}&{{{\textbf{F}}_{E32}}} \end{array}} \right] , \hspace{5.0pt}{{\textbf{F}}_{E31}},{{\textbf{F}}_{E32}} \in {{\mathbb {C}}^{2{N_E} \times {N_E}}}} \end{aligned}$$

So we have

32 $$\begin{aligned} {{\text {tr}}({{\textbf{S}}_{E3}}{{\textbf{M}}_{E3}}) = {\left\| {\begin{array}{*{20}{c}} {{\text {vec}}({{\textbf{F}}_{E31}})} \\ {{\text {vec}}({{\textbf{F}}_{E32}})} \\ {{\text {vec}}(\sigma _E^{ - 1}{{\textbf{F}}_{E31}}{{\textbf{H}}_{RE,1}}{\textbf{V}})} \\ {{\text {vec}}(\sigma _E^{ - 1}{{\textbf{F}}_{E32}}{{\textbf{H}}_{SE,2}}{\textbf{A}})} \\ {{\text {vec}}(\sigma _E^{ - 1}{{\textbf{F}}_{E32}}{{\textbf{H}}_{RE,2}}{\textbf{G}}{{\textbf{H}}_{RR,1}}{\textbf{V}})} \\ {{\text {vec}}(\sigma _E^{ - 1}\sigma _R^{}{{\textbf{F}}_{E32}}{{\textbf{H}}_{RE,2}}{\textbf{G}})} \\ {{\text {vec}}(\sigma _E^{ - 1}{{\textbf{F}}_{E31}}{{\textbf{H}}_{SE,1}}{\textbf{W}})} \\ {{\text {vec}}(\sigma _E^{ - 1}{{\textbf{F}}_{E32}}{{\textbf{H}}_{RE,2}}{\textbf{G}}{{\textbf{H}}_{SR,1}}{\textbf{W}})} \end{array}} \right\| ^2}} \end{aligned}$$

Focusing on the uncertain CTF, (30) can be rewritten as follows.

33 $$\begin{aligned} {{{\phi _D} = {{\bar{\phi }} _D} + \underbrace{\sum \limits _j^{} {{{\varvec{\Omega }}_{Dj}}{\text {vec}}({{\mathbf {\Delta }}_j})} }_{{{\mathbf {\Delta }}_D}} + \underbrace{\sum \limits _k^{} {{\alpha _k}{\text {vec}}({{\mathbf {\Delta }}_{k1}}){\text {ve}}{{\text {c}}^{H}}({{\mathbf {\Delta }}_{k2}})} }_{{{\widetilde{\mathbf {\Delta }}}_D}}}} \end{aligned}$$

where the identity $\text {vec}(\textbf{ABC})=\left( {{\textbf{C}}^{T}}\otimes \textbf{A} \right) \text {vec}\left( \textbf{B} \right)$ is applied. ${{\mathbf {\Delta }}_D}$ and ${{{\widetilde{\mathbf {\Delta }}}_D}}$ is the linear part and the quadratic part of the CTF uncertainty, respectively. Actually the quadratic part is negligible. Then, we only consider asymptotic form of ${\phi _D}$ as

34 $$ \phi _{D}  = \bar{\phi }_{D}  + \underbrace {{\sum\limits_{j} {\varvec\Omega _{{Dj}} {\text{vec}}({\mathbf{\Delta }}_{j} )} }}_{{{\mathbf{\Delta }}_{D} }} $$

where

35 $$ \varvec{\Omega} _{{DSR,1}}  = \left[ {\begin{array}{*{20}c}    {{\mathbf{W}}^{T}  \otimes {\mathbf{F}}_{D} {\mathbf{D}}_{D} \overline{{\mathbf{H}}} _{{RD,2}} {\mathbf{G}}}  \\    {\mathbf{0}}  \\    {\mathbf{0}}  \\    {\mathbf{0}}  \\   \end{array} } \right],{ \varvec\Omega}_{{DSD,2}}  = \left[ {\begin{array}{*{20}c}    {({\mathbf{G}}\overline{{\mathbf{H}}} _{{SR,1}} {\mathbf{W}})^{T}  \otimes {\mathbf{F}}_{D} {\mathbf{D}}_{D} }  \\    {({\mathbf{G}}\overline{{\mathbf{H}}} _{{RR,1}} {\mathbf{V}})^{T}  \otimes {\mathbf{F}}_{D} {\mathbf{D}}_{D} }  \\    {\sigma _{R} {\mathbf{G}}^{T}  \otimes {\mathbf{F}}_{D} {\mathbf{D}}_{D} }  \\    {\mathbf{0}}  \\   \end{array} } \right], \varvec{\Omega} _{{DRR,1}}  = \left[ {\begin{array}{*{20}c}    {\mathbf{0}}  \\    {{\mathbf{V}}^{T}  \otimes {\mathbf{F}}_{D} {\mathbf{D}}_{D} \overline{{\mathbf{H}}} _{{RD,2}} {\mathbf{G}}}  \\    {\mathbf{0}}  \\    {\mathbf{0}}  \\   \end{array} } \right] $$

For other situations and for the constraint ${\textrm{tr}}({({\textbf{G}}{{\textbf{H}}_{SR,1}}{\textbf{W}})^K}+{({\textbf{G}}{{\textbf{H}}_{RR,1}}{\textbf{V}})^K} + \sigma _R^2{{\textbf{G}}^K})\le {P_R}$, similiar formulas can be obtained through the same method. While the power constraint does not involve any quadratic part of the CTF uncertainty, so the original problem constraints are not relaxed.

With (30) and (34) and by exploiting the Schur complement lemma^75^, (28) can be rewritten as matrix inequality .

36 $$\begin{aligned} \left[ {\begin{array}{*{20}{c}} {{\beta _D}}&{}{{{{\bar{\phi }} }_D}^H}\\ {{{{\bar{\phi }} }_D}}&{}{\textbf{I}} \end{array}} \right] \succ - \left[ {\begin{array}{*{20}{c}} 0&{}{{{\varDelta }}_D^H}\\ {{{{\varDelta }}_D}}&{}{\textbf{0}} \end{array}} \right] \end{aligned}$$

The constraint (36) still contains the uncertainty ${{\mathbf {\Delta }}_{D}}$. The sign-definiteness lemma is applied to eliminate this uncertainty.

Lemma 2^76^: Given a Hermitian matrix $\textbf{A}$ and arbitrary matrices pair $\left\{ {{\textbf{P}}_{i}},{{\textbf{Q}}_{i}} \right\} ,\;i\in \left\{ 1,2,\ldots ,N \right\}$, the semi-infinite Linear Matrix Inequality (LMI)

37 $$\begin{aligned} \textbf{A}\succ \sum \limits _{i}^{N}{\left( \textbf{P}_{i}^{H}{{\textbf{Y}}_{i}}{{\textbf{Q}}_{i}}+\textbf{Q}_{i}^{H}\textbf{Y}_{i}^{H}\textbf{P}_{i}^{{}} \right) },\;\left\| {{\textbf{Y}}_{i}} \right\| \le {{\delta }_{i}} \end{aligned}$$

holds if and only if there exist nonnegative real numbers ${{\lambda }_{1}},{{\lambda }_{2}},\ldots ,{{\lambda }_{N}}$ such that

38 $$\begin{aligned} \left[ \begin{array}{llll} \textbf{A}-\sum \nolimits _{i=1}^{N}{{{\lambda }_{i}}\textbf{Q}_{i}^{H}\textbf{Q}_{i}^{{}}} &{} -{{\delta }_{1}}\textbf{P}_{1}^{H} &{} \cdots &{} -{{\delta }_{N}}\textbf{P}_{N}^{H} \\ -{{\delta }_{1}}\textbf{P}_{1}^{{}} &{} {{\delta }_{1}}\textbf{I} &{} \cdots &{} \textbf{0} \\ \vdots &{} \vdots &{} \ddots &{} \vdots \\ -{{\delta }_{N}}\textbf{P}_{N}^{{}} &{} \textbf{0} &{} \cdots &{} {{\delta }_{N}}\textbf{I} \\ \end{array} \right] \succ \textbf{0} \end{aligned}$$

39 $$ h \triangleq 2\log \left| {{\mathbf{F}}_{D} } \right| - \beta _{D}  + 2\log \left| {{\mathbf{F}}_{{E1}} } \right| - \beta _{{E1}}  + 2\log \left| {{\mathbf{F}}_{{E2}} } \right| - \beta _{{E2}}  + 2\log \left| {{\mathbf{F}}_{{E3}} } \right| - \beta _{{E3}}  $$

Appropriately choose the parameters as below

40 $$\begin{aligned} {{{{\textbf{A}}_D} = \left[ {\begin{array}{*{20}{c}} {{\beta _D}}&{}{{\bar{\phi }} _D^H}\\ {{{{\bar{\phi }} }_D}}&{}{\textbf{I}} \end{array}} \right] ,{{\textbf{Q}}_{D1}},{{\textbf{Q}}_{D2}},{{\textbf{Q}}_{D3}} = [ - 1{\textbf{0}}],{{\textbf{P}}_{D1}} = \left[ {\begin{array}{*{20}{c}} {\textbf{0}}&{{{\varvec{\Omega }}}_{DSR,1}^H} \end{array}} \right] ,{{\textbf{P}}_{D2}} = \left[ {\begin{array}{*{20}{c}} {\textbf{0}}&{{{\varvec{\Omega }}}_{DSD,2}^H} \end{array}} \right] ,{{\textbf{P}}_{D3}} = \left[ {\begin{array}{*{20}{c}} {\textbf{0}}&{{{\varvec{\Omega }}}_{DRR,1}^H} \end{array}} \right] }} \end{aligned}$$

Apply lemma 2 and insert (40) to (36) , and we have

41 $$\begin{aligned} \left[ \begin{array}{llll} \left[ \begin{array}{llll} {{\beta }_{D}}\!\!-\!\!{{\lambda }_{D1}}\!\!-\!\!{{\lambda }_{D2}}\!\!-\!\!{{\lambda }_{D3}} &{} {{{\bar{\phi }}}_{D}}^{H} \\ {{{\bar{\phi }}}_{D}} &{} \textbf{I} \\ \end{array} \right] &{} {{{\theta }}}_{D}^{H} \\ {{{\theta }}_{D}} &{} \text {diag}({{\lambda }_{D1}}\textbf{I} ,{{\lambda }_{D2}}\textbf{I},{{\lambda }_{D3}}\textbf{I}) \\ \end{array} \right] \succ 0 \end{aligned}$$

where ${{{\theta }}_{D}}=-{{[{{\delta }_{DSR,1}}{{\textbf{P}}}_{D1}^{T},{{\delta }_{DSD,2}}{{\textbf{P}}}_{D2}^{T},{{\delta }_{DRR,1}}{{\textbf{P}}}_{D3`}^{T}]}^{T}}$. Similarly, other inequalities $\text {tr}({{\textbf{S}}_{i}}{{\textbf{M}}_{i}})\le {{\beta }_{i}}$ can be transfered into the same form as following.

42 $$\begin{aligned} \left[ {\begin{array}{*{20}{c}} {\left[ {\begin{array}{*{20}{c}} {{\beta _i} - {{\sum \limits _{k = l}^j {{\lambda _k}} }}}&{}{{\bar{\phi }} _i^H}\\ {{{{\bar{\phi }} }_i}}&{}{\textbf{I}} \end{array}} \right] }&{}{{{\theta }}_i^H}\\ {{{{\theta }}_i}}&{}{\left[ {{\lambda _l}{\textbf{I}}, \cdots ,{\lambda _j}{\textbf{I}}} \right] } \end{array}} \right] \succ 0 \end{aligned}$$

With all components, the problem is equivalent to

43 $$\begin{aligned} {{\mathop {\max }\limits _{{\textbf{W}},{\textbf{V}},{\textbf{A}},{\textbf{G}},{{\textbf{F}}_i} \succ 0,{{\textbf{D}}_i},{\lambda _i} \ge 0,{\beta _i}} {\hspace{0.55542pt}} {h}({\textbf{W}},{\textbf{V}},{\textbf{A}},{\textbf{G}},{\textbf{F}}_{\textbf{i}},{\textbf{D}}_{\textbf{i}},{\lambda _i},{\beta _i})}}, \quad {\textrm{s}}{\mathrm{.t}}{{.\;(16),\; (40),\;(41)}} \end{aligned}$$

where the function $h(\textbf{W},\textbf{V},\textbf{A},\textbf{G},{\textbf{F}}_{\textbf{i}},{\textbf{D}}_{\textbf{i}},{{\lambda }_{i}},{{\beta }_{i}})$ is defined in (39).

Although (43) is still non-convex. However, it is convex with respective to ${{\textbf{F}}_{i}}$ or any one in $\textbf{W},\textbf{V},\textbf{A},\textbf{G},{{\textbf{D}}_{\textbf{i}}}$. As a result, (43) can be solved via BCD algorithm as below.

**Algorithm 1:**
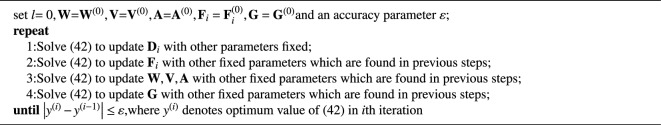
Jamming precoding scheme to solve problem (43).

The variables to be optimized in the optimization problem are divided into the following groups.

44 $$\begin{aligned} {\Phi _0} = \{ {\lambda _i},{\beta _i}\} ,{\Phi _1} = \left\{ {{{\textbf{D}}_i},{\Phi _0}} \right\} ,{\Phi _2} = \left\{ {{{\textbf{F}}_i},{\Phi _0}} \right\} ,{\Phi _3} = \left\{ {{\textbf{W}},{\textbf{V}},{\textbf{A}},{\Phi _0}}, \right\} ,{\Phi _4} = \left\{ {{\textbf{G}},{\Phi _0}} \right\} \end{aligned}$$

By optimizing the problem in order ${\Phi _1} \rightarrow {\Phi _2} \rightarrow {\Phi _3} \rightarrow {\Phi _4}$, we denote $y_k^{\left( i \right) }$ as the optimal value optimized to ${\Phi _k}$ in the *i*th iteration. Since the variables to be optimized always meet the constraints in the optimization process, the optimal value will not decrease :

45 $$\begin{aligned} y_{}^{\left( i \right) } = y_4^{\left( i \right) } \ge y_3^{\left( i \right) } \ge y_2^{\left( i \right) } \ge y_1^{\left( i \right) } \ge y_{}^{\left( {i - 1} \right) } = y_4^{\left( {i - 1} \right) } \ge y_3^{\left( {i - 1} \right) } \ge y_2^{\left( {i - 1} \right) } \ge y_1^{\left( {i - 1} \right) } \cdots \end{aligned}$$

Obviously with constraints (16), the secrecy rate is bounded and the objective function value increases in each iteration, which proves the convergence.

## Simulation and results

In the simulation parts, the statistical MIMO PLC channels are generated by formula (1) in reference^71^, and the specific parameters are shown in Table 2. And the noises in PLC are also modeled as a Bernoulli–Gaussian impulsive noise.

**Table 2:** Parameters of the PLC channels^71^.

Path loss parameter	Model	Parameters
${A_{{\text {dB}}{\hspace{0.55542pt}} {\text { }}S{\hspace{0.55542pt}} 1,D1}}$	${\mathcal {N}}(\mu _{A},\sigma _{A})$	$$\begin{array}{*{20}{c}} {{\mu _A}{\hspace{0.55542pt}} = {\hspace{0.55542pt}} - 50.1{\hspace{0.55542pt}} \;{\text {dB}}} \\ {{\sigma _A}{\hspace{0.55542pt}} = {\hspace{0.55542pt}} 15.6\;{\text {dB}}} \end{array}$$
$$\begin{aligned} {A_{{\text {dB }}Sm,Dn}}{\hspace{0.55542pt}} \\ m \in [1,2,3] \\ \end{aligned} $$	${A_{{\text {dB}}{\hspace{0.55542pt}} {\hspace{0.55542pt}} Sm,Dn}} = {A_{{\text {dB}}{\hspace{0.55542pt}} {\hspace{0.55542pt}} S1,D1}} + \mathcal {N}(0,{\sigma _{Sm,Dn}})$	$$\left[ {{\sigma _{Sm}},Dn} \right] = \left[ {\begin{array}{*{20}{c}} 0&{}{5.1}&{}{3.8} \\ {2.9}&{}{5.7}&{}{5.2} \\ {6.6}&{}{7.8}&{}{6.9} \\ {4.6}&{}{5.9}&{}{5.1} \end{array}} \right] {\text {dB}}$$
$A_{\textrm{dB}\,S4,Dn}$	${A_{{\text {dB}}{\hspace{0.55542pt}} {\hspace{0.55542pt}} S4,Dn}} = 0.5 \times {A_{{\text {dB}}{\hspace{0.55542pt}} {\hspace{0.55542pt}} S1,D1}} - 30 + \mathcal {N}(0,{\sigma _{S4,Dn}})$	
${a_0}$	${E_{{\text {shift}}}}({\mu _{{a_0}}},{\delta _{{a_0}}})$	$$\begin{aligned} {\mu _{{a_0}}} = 1.04 \times {10^{ - 2}} \\ {\delta _{{a_0}}} = - 6.7 \times {10^{ - 3}} \\ \end{aligned} $$
${a_1}$	Constant	$a_{1}=4\times 10^{-10}$
*K*	$\mathcal {W}(\alpha _{K},\beta _{K})$	$$\begin{array}{c}{{\alpha _{K}=5.7\times 10^{-2}}}\\ {{\beta _{K}=57.7}}\end{array}$$
$L_{max}$	Constant	$L_{max}=800\,\textrm{m}$
$\Lambda$	Constant	$\Lambda =0.2\,\textrm{m}^{-1}$

In this section, numerical results are presented to prove the effectiveness of precoding jamming scheme in terms of average secrecy rate. In this part, without specific definition, we consider ${N_S} = {N_R} = {N_D} = {N_E} = N = 2$. Besides, for the simplicity, the CTF uncertaninty bound ${{\delta _{ij,k}}}$ are related to corresponding determinant of mean CTF with one certain coefficient, or ${\delta _{ij,k}} = \mu \left\| {\overline{\textbf{H}_{ij,k}}} \right\|$. Apparently, it accords with the natural assumption that CTF with larger determinant tends to be more uncertain.

**Figure 3:**
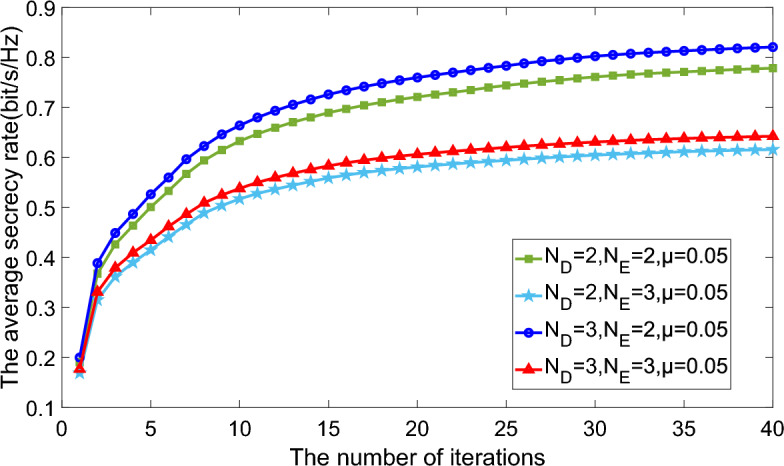
Average secrecy rate versus numbers of iterations comparison of different ports number and CTF uncertainty.

**Figure 4:**
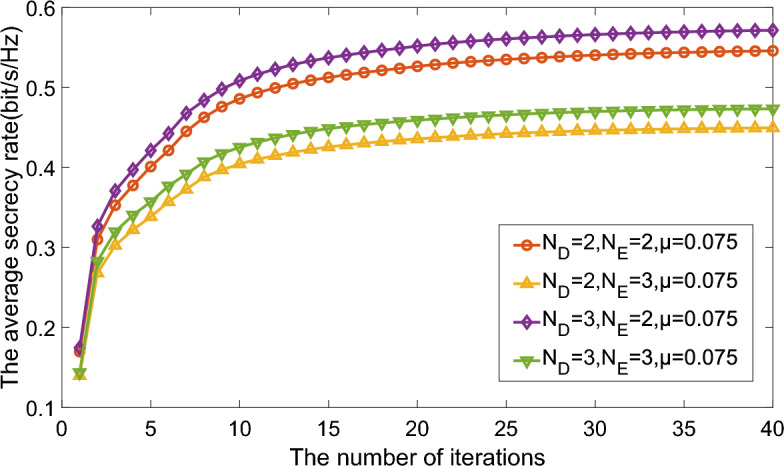
Average secrecy rate versus numbers of iterations comparison of different ports number and CTF uncertainty.

Figures 3 and 4 illustrate the relationship between the average secrecy rate and the number of iterations, assuming power constraints ${P_S} = {P_R} = P = 10 \,{\text {dB}}$. Notably, the average secrecy rate consistently stabilizes after approximately 40 iterations. This indicates that heightened CTF uncertainty detrimentally impacts the secrecy rate. Moreover, the proposed approach exhibits superior performance with an increased number of legitimate user ports and a reduced number of eavesdropper ports. This distinction becomes particularly pronounced in scenarios characterized by larger CTF uncertainty. In essence, the number of ports directly correlates with the capacity for receiving or intercepting information.

**Figure 5:**
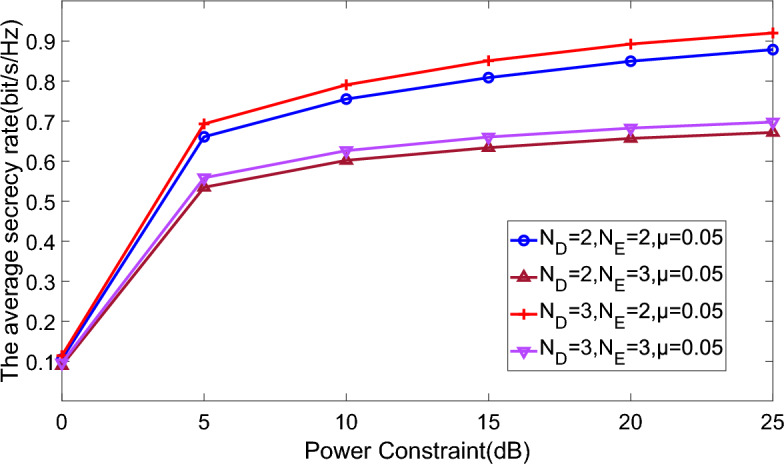
Average secrecy rate versus power constraint comparison of different ports number and CTF uncertainty.

**Figure 6:**
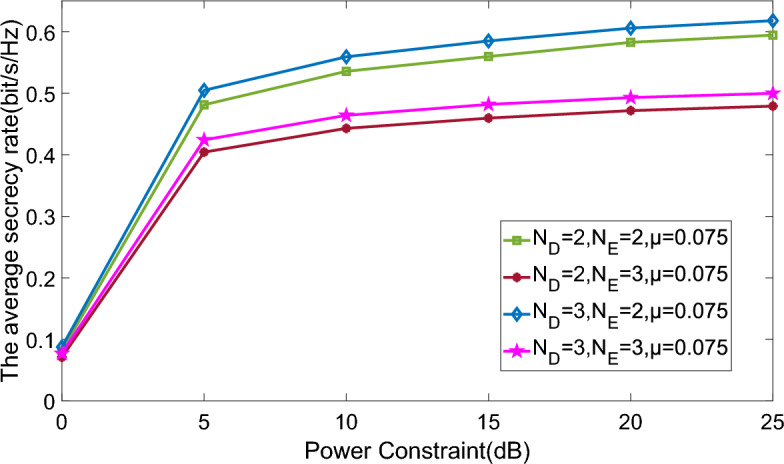
Average secrecy rate versus power constraint comparison of different ports number and CTF uncertainty.

We examine the characteristics of the proposed scheme under varying transmit power levels in Figs. 5 and 6. The analysis reveals an increase in the average secrecy rate as the transmit power is raised. However, beyond a transmission power of 10 dB, especially in scenarios with more eavesdropper ports and increased CTF uncertainty, the secrecy rate experiences only marginal improvement. This is attributed to the fact that elevating transmit power enhances not only the capacity of legitimate users but also that of eavesdroppers colluding to boost their eavesdropping rate. Consequently, this simultaneous enhancement leads to only a slight alteration in the overall secrecy rate. Additionally, when the numbers of legitimate user ports and eavesdropper ports both increase from 2 to 3, there is a notable decrease in the average secrecy rate. This observation suggests that in collusion scenarios, the expansion of the eavesdroppers’ port number has a more substantial impact on the PLC system than the growth in the number of legitimate users’ ports.

**Figure 7:**
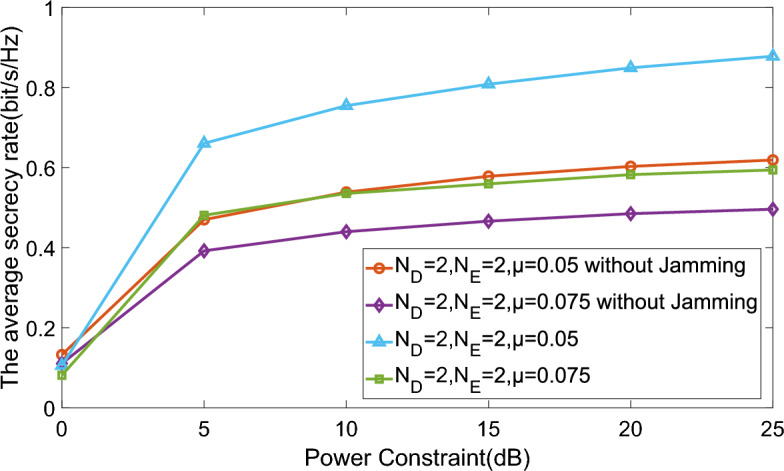
Average secrecy rate versus power constraint comparison of different schemes.

We assess the influence of jamming by showcasing the numerical outcomes of our proposed schemes alongside a comparable one lacking jamming signals in Fig. 7. This figure shows improvements achieved by the proposed algorithm compared to the traditional one. As shown in Fig. 7, the proposed algorithm achieves higher secure rates than the traditional algorithm when $\mu =0.05$ or $\mu =0.075$, and the advantage increases with increasing power. Specifically, when $\mu =0.05$ and the transmission power is 20 dB, the secure rate of the proposed algorithm can reach 0.85 bit/s/Hz, while the traditional algorithm achieves 0.61 bit/s/Hz. In contrast to the jamming-free scheme, our proposed approach demonstrates superior performance, particularly regarding the average secrecy rate, especially under conditions of lower CTF uncertainty and increased transmit power. This suggests that, to a certain degree, jamming has the capability to disrupt the interception efforts of eavesdroppers, even in scenarios characterized by higher CTF uncertainty.

**Figure 8:**
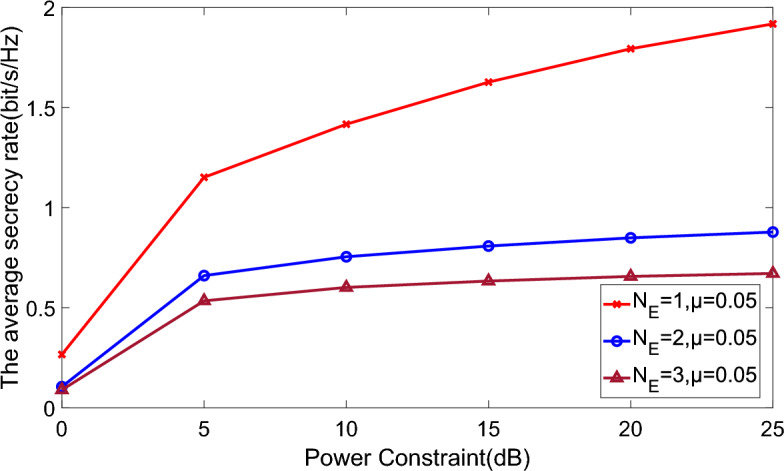
Average secrecy rate versus different eavesdropping ports number.

Figure 8 depicts how the ports of eavesdroppers affect the PLC system, where ${N_S} = {N_R} ={N_D}= 2$. It suggests the ability of eavesdroppers increases with the growth of their ports, especially in few ports.

## Conclusion

The paper introduces a precoding jamming scheme aimed at bolstering the security of AF relay-aided PLC systems when faced with the challenge of multiple colluding eavesdroppers, while also considering CTF uncertainty. The numerical results unequivocally establish the superiority of our proposed scheme compared to a jamming-free alternative. Notably, the effectiveness of the proposed scheme is underscored, especially in scenarios characterized by elevated CTF uncertainty.

## Data Availability

The datasets generated and/or analysed during the current study are available in the github repository, https://github.com/zilongmi/Jamming-Precoding-in-AF-Relay-aided-PLC-Systems-with-Multiple-Eavessdroppers.
